# Cancer Chemotherapy Specific to Acidic Nests

**DOI:** 10.3390/cancers9040036

**Published:** 2017-04-20

**Authors:** Hiroshi Kobayashi

**Affiliations:** Graduate School of Pharmaceutical Sciences, Chiba University, Chiba 260-8675, Japan; hiroshi@faculty.chiba-u.jp; Tel.: +81-263872529

**Keywords:** chemotherapy, acidosis of cancer nests, statins, cantharidin, manumycin A, ionomycin, acidosis-dependent gene expression

## Abstract

The realization of cancer therapeutics specific to cancer cells with less of an effect on normal tissues is our goal. Many trials have been carried out for this purpose, but this goal is still far from being realized. It was found more than 80 years ago that solid cancer nests are acidified, but in vitro studies under acidic conditions have not been extensively studied. Recently, in vitro experiments under acidic conditions were started and anti-cancer drugs specific to acidic areas have been identified. Many genes have been reported to be expressed at a high level under acidic conditions, and such genes may be potent targets for anti-cancer drugs specific to acidic nests. In this review article, recent in vitro, in vivo, and clinical achievements in anti-cancer drugs with marked efficacy under acidic conditions are summarized, and the clinical use of anti-cancer drugs specific to acidic nests is discussed.

## 1. Introduction

Over the past 80 years, many researchers have tried to measure the pH of tumor tissues [1], and it is now accepted that solid cancer nests are generally acidified [2,3]. The acidosis is mediated via the acceleration of glycolysis, which is called the Warburg effect [4]. This acceleration is mainly induced by the limited supply of oxygen [2]. In another report, an increase in glycolytic activity was observed with a sufficient supply of oxygen in solid cancer nests [4].

Drugs with increased efficacy to inhibit cancer cell proliferation under acidic conditions have been identified recently. Such drugs may have less effects on normal tissues, whose pH is slightly alkaline. However, their clinical application is still limited. In this review, recent achievements in anti-cancer drugs with marked efficacy under acidic conditions are summarized, and the clinical use of such acidosis-dependent drugs is discussed.

## 2. Metabolic Pathways Working under Acidic Conditions

The effect of acidosis on cancer cell functions has not been well discussed until recently. One reason may be that the pH change in cancer nests is often less than 1 pH unit. Another may be the argument that the pH in intracellular spaces is not affected by the acidification of the surroundings within this narrow range of pH change. Cytosolic acidification, however, was observed in cancer cells with a decrease in the pH of the culture medium [5,6]. Cytosolic pH values were 7.4 and 6.9 in media with a pH of 7.4 and 6.5, respectively [5]. In another report, cytosolic pH values were 7.4 and 6.8 in media with a pH of 7.4 and 6.2, respectively [6]. These data suggest that the pH homeostatic capacity of the cytosolic space is not strong enough to maintain a constant cytosolic pH. Thus, the cytosol may be acidified in acidic cancer nests.

A question that arises is whether cytosolic acidification affects cellular metabolism within the narrow pH change. Since all enzymes mediating cellular metabolism have pH-dependent activity, cytosolic acidification affects the activities of some metabolic pathways. The change of 1 pH unit corresponds to a 10-fold change in the proton concentration, which should affect enzyme activities. When a metabolic pathway declines at acidic pH, it would be beneficial for an alternative pathway to work to compensate the decline. It is well known that bacteria have metabolic pathways that function under acidic conditions [7]. These previous insights led us to consider that mammals also have alternative systems working in acidic diseased areas, such as cancer nests, inflammation loci, and areas of infarction.

Our research group investigated the expression of 24,000 genes in cancer cells cultured in media at pH 7.5 and 6.7, and found that the mRNA levels of approximately 700 genes were increased at the acidic pH [8]. Among the 700 genes, 24% encoded signal proteins, external ligands, and regulatory proteins. Many of these proteins may participate in the promotion and regulation of cell proliferation. These results suggest that a large number of proteins are working preferentially under acidic conditions.

Lao et al. [9,10] identified a protein whose expression level at pH 6.3 was the same as that at pH 7.4 in Chinese hamster ovary cells, but this protein was only essential for growth under acidic conditions. The same protein was found in mammalian cells [11]. These results suggest that mammalian cells have proteins required for growth at an acidic pH, although their synthesis is constitutive, besides proteins encoded by genes whose expression increases at an acidic pH.

Recently, an increased expression of OCT-4 was observed in fibroblasts growing in an acidic medium at pH 6.5 for 7 days [12]. Hjelmeland et al. [13] reported increased expressions of OCT-4, Olig2, and Nanog in glioma stem cells treated at pH 6.5 for 6 days. The measurement of gene expression under acidic conditions is still limited. Without having to wait for future studies, the present data suggest that mammalian cells contain a high number of acidosis-dependent genes.

## 3. Acidosis-Dependent Anti-Cancer Drugs

The investigation of cancer-specific targets has been focused on, which may be useful for anti-cancer chemotherapy with less effects on normal tissues. Cancer-specific targets vary in different organs and, more seriously from a treatment perspective, they vary among individuals. The investigation of cancer-specific targets is still in progress. The acidification of solid cancer nests provides a clue that molecules involved in metabolic pathways working preferentially at an acidic pH may be potent targets for chemotherapy against cancers, because such molecules would be less functional in normal tissues, whose pH is slightly alkaline.

Fukamachi et al. [14] screened approximately 280 compounds including enzyme inhibitors using HeLa cells in media at pH 7.7–7.4 and 6.7–6.6, and two compounds, lovastatin and cantharidin, were found to inhibit cell growth preferentially at an acidic pH. In contrast, the inhibitory efficacies of vinblastine sulfate, paclitaxel, aclarubicin, aphidicolin, trichostatin A, and 17-AAG decreased at an acidic pH (Table 1). The efficacies of 14 compounds were almost the same at both pH values (Table 1). Others showed no inhibition of HeLa cell proliferation at 1 μM in alkaline or acidic media. Manumycin A and ionomycin preferentially showed an inhibitory effect at an acidic pH in mesothelioma [14] and synovial cells [15], respectively.

The question is whether extracellular pH, cytosolic pH, or both are responsible for high efficacy of acidosis-dependent drugs. The target molecules of statins, cantharidin, and manumycin A have been reported to be cytosolic enzymes, as described below, and the accumulation of statins was almost the same at both pH 6.7 and 7.4 [16]. These results suggest that cytosolic acidification is the main factor to improve the efficacy of these drugs.

### 3.1. Statins

Lovastatin, simvastatin, and atorvastatin were shown to inhibit cell proliferation of all cancer cells tested preferentially in medium at pH 6.7 [14,15]. Statins inhibit mevalonate pathways and attenuate the level of farnesyl diphosphate, which is used for two metabolic pathways; syntheses of cholesterol and geranylgeranyl diphosphate [15]. Geranylgeranyl diphosphate and farnesyl diphosphate are used for protein prenylation [15]. Prenylated small G proteins, such as Ras, Rho, Rab, Arf, and Ran [17], move to membranes and work as proteins for signal pathways [18]. Therefore, statins decrease the levels of cholesterol and prenylated proteins. The effect of statins on the blood level of cholesterol has been researched, and these drugs have been developed as agents against hyperlipidemia. The decrease in protein prenylation was shown to be the main mechanism for the inhibition of cancer cell proliferation at an acidic pH in vitro [14].

### 3.2. Cantharidin

Cantharidin is an active compound of the traditional Chinese medicine mylabris, prepared from the Chinese blister beetle, and mylabris has been used to cure cancer for more than 700 years in China [19]. Its clinical application is still limited, although the effectiveness of cantharidin has been reported [20]. The main problem is toxicity, especially that causing liver injuries [21]. Cantharidin inhibited the proliferation of mesothelioma at 1 μM in an acidic medium [14], and ED50 in Ehrlich ascites carcinoma was approximately 130 μM in an alkaline medium used conventionally [22]. In a mouse model, the i.v. administration of 1 mg/kg every 3 days was shown to be effective [23]. When sodium cantharidin was administered (0.5 g/day) to patients with gastric cancer who received mainly fluoropyrimidine-based chemotherapy, no significant toxicity was observed [24]. Similar results were reported in patients with breast cancer receiving combined chemotherapy with cyclophosphamide, epirubicin, and docetaxel [25]. These results suggest that cantharidin can be used alone as an anti-cancer medicine. Vitamin C administration was reported to attenuate the liver injuries induced by cantharidin in mice [21], but the effect of vitamin C on anti-cancer therapy with cantharidin remains unclear. Derivatives of this drug have been synthesized to reduce the toxicity [26]. The target of cantharidin was reported to be protein phosphatases, PP1 and PPA2 [27], but the mechanism leading to the inhibition of cell proliferation at an acidic pH is unknown.

### 3.3. Manumycin A

Manumycin A was isolated from *Streptomyces* [28], and inhibits the prenylation of small G-protein [29]. The administration of manumycin A prevented the growth of triple-negative breast cancers, in which the expressions of estrogen, progesterone, and HER2 receptors were negative, in nude mice [29]. Manumycin A was also shown to increase the efficacy of other anti-cancer drugs in animal models [28]. To the best of the author’s knowledge, no clinical trial using manumycin A has been reported. The main reason for the lack of clinical application would be that the in vitro efficacy to inhibit cancer cell growth is weak under alkaline conditions [14,28].

### 3.4. Ionomycin

Ionomycin was reported to inhibit cell proliferation much more markedly at an acidic pH [15]. Ionomycin increases membrane permeability to calcium ions and induces cell death [30]. It is unclear why the effect of ionomycin on cell death increases at an acidic pH. The intracellular calcium ion concentration was higher in an acidic medium than that in an alkaline medium [31]. The increased cytotoxicity of ionomycin at an acidic pH might be related to the increase in the intracellular calcium ion concentration at an acidic pH.

### 3.5. Doxorubicin

Doxorubicin did not show a clear difference in the inhibition of HeLa cell growth between alkaline and acidic pH values, but the cell shape changed at an acidic pH at 1 μM [14], suggesting that cell growth could be inhibited at a higher concentration under acidic conditions, but no data confirming this have been reported.

## 4. Anti-Cancer Drugs Whose Efficacy Decreases at an Acidic pH

Anti-cancer drugs with marked efficacy at an acidic pH are still limited. In the case of many anti-cancer drugs, cancer cells become tolerant to chemotherapy. Multi-drug resistance was induced by acidosis in rat prostate cancer cells via increasing the activity of p-glycoprotein [32]. Uptakes of mitoxantrone and topotecan decreased in vitro at an acidic pH [5]. Reduced drug accumulation may be a reason for the decrease in chemotherapeutic efficacy at an acidic pH. Cancer progression occurs frequently with inadequate angiogenesis, resulting in the limited supply of drugs from blood vessels [2]. This is a reason for the decrease in the efficacy of anti-cancer drugs in vivo. The mRNA levels of approximately 850 genes were decreased at an acidic pH among 24,000 genes tested in mesothelioma [8]. The functional decline of the target molecules may cause resistance to anti-cancer drugs in an acidic environment.

## 5. Proposal for Clinical Usage of Statins

As described above, the clinical protocol for the use of statins against cancers has not been established. Kawata et al. [33] reported that pravastatin promoted prolonged survival in patients with advanced hepatocellular carcinoma. Since this report, promising results have been reported with statin use in patients bearing a wide array of cancer types, while some large meta-analyses have not found an association between statin use and outcomes in cancer patients (for reviews, see [34,35]). Statins have been prescribed as anti-hyperlipidemia agents. Defined daily doses (DDDs) of statins recommended by the WHO Collaborating Centre for Drug Statistics Methodology (WHOCC) are 20 to 60 mg (Table 2). These values were determined based on the effective concentration for decreasing the blood cholesterol level. The main mechanism for the anti-cancer effect of statins is the attenuation of protein prenylation, not the inhibition of cholesterol synthesis [14]. Therefore, the recommended DDDs may not be sufficient for therapy against cancers. In fact, the data obtained in mouse models demonstrated that the effective concentration of statins to attenuate cancer progression was 10–50 mg/kg/day administered orally (Table 2) [36,37,38,39,40,41,42,43]. The estimated dose of simvastatin for oral administration in a human patient whose weight is 60 kg is 3000 mg/day, which is 100-fold higher than the DDD range. In the case of lovastatin, 600–960 mg/day of lovastatin are estimated for a human patient whose weight is 60 kg, while the DDD is 45 mg. Forty mg/kg/day of fluvastatin in mice corresponds to 2400 mg/day for oral administration in a human patient whose weight is 60 kg, which is 40-fold higher than the DDD. The administration of statins at a low dose may have the opposite effects in clinical investigations.

**Figure 1:**
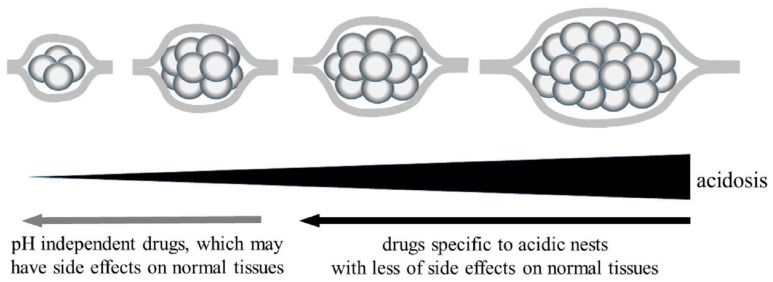
Proposal for chemotherapy using anti-cancer drugs specific to acidic nests.

Since statins are effective under acidic conditions, their efficacy may be dependent on the acidosis of cancer nests. Acidosis-dependent anti-cancer drugs are less effective in areas insufficiently acidified, such as at an early stage, in blood, or in cancer nests where angiogenesis is highly active. In fact, the negative effect of statins has been reported in patients with early-stage hepatocellular carcinoma [44], follicular lymphoma [45], and prostate cancer after radical prostatectomy [46]. This is another reason for the opposite results in clinical examinations. On the basis of these investigations, the author would like to propose a clinical protocol whereby statins are first used, and then other methods, such as excision, radiation, immunotherapy, and chemotherapy with conventional drugs whose efficacy is not dependent on the pH, are applied after cancer nests have been reduced in size (Figure 1). The strategy to use conventional drugs in this stage would be advantageous, because the drug dosage can be reduced in small cancer nests to ameliorate the side effects in normal tissues.

Chemotherapy specific to acidic cancer nests has been argued to have weaker effects on cells in normal tissues, whose pH is slightly alkaline. A serious side effect of conventional drugs is the dysfunction of the immune system. Statins inhibited the proliferation of immune cells at an acidic pH, but the cytotoxicity of statins against immune cells was very low at pH 7.5 [47]. This report suggests that statins have less of an effect on the immune system at a level sufficient for the attenuation of cancer progression in acidic areas. Statins are now prescribed as drugs against hyperlipidemia, and no dysfunction of the immune system caused by statins has been reported. Statins were reported to increase incident diabetes, but the adjusted hazard ratio was 1.04 to 1.17 [48]. The risk of a major cardiovascular event was less than 1% in patients treated with statins [49]. Statin administration at a high dose may cause hypocholesterolemia. Although no clinical protocol for treatment against hypocholesterolemia induced by drugs has been reported, it might be cured by a special diet, as reported in hypobetalipoproteinemia and abetalipoproteinemia caused by genetic mutation [50]. These observations indicate that the side effects of statins on normal tissues are not serious.

## 6. Measurements of Acidity in Cancer Nests

The in vivo measurement of acidity in cancer nests would help us use acidosis-dependent anti-cancer drugs more efficiently. Recently, new in vivo methods to measure the pH of cancer areas have been developed in animal models [51,52]. As described above, many genes are expressed at high levels under acidic conditions, and the measurement of the degraded materials of mRNAs encoded by such genes in blood might be useful to understand the acidity of cancer nests. The genes, whose expression increased with the acidification of cancer nests, have been shown to be different in different organs [53]. The measurement of the acidity is important not only for evaluation of the cancer progression, but also to decide on whether acidosis-dependent anti-cancer drugs are applicable.

## 7. Exploitation of Other Drugs Specific to Acidic Cancer Nests

Four compounds have been demonstrated to show anti-cancer activity preferentially under acidic conditions. A question is whether other acidosis-dependent anti-cancer drugs can be exploited in the future. Many genes were shown to be expressed at high levels at an acidic pH [8]. Among the genes whose expression increased at an acidic pH, 24% can be argued to encode factors for supporting cell proliferation under acidic conditions [8]. Medium acidosis increased the phosphorylation of AKT and ERK, and induced nuclear translocation of NF-κB [54]. These observations suggest the possibility of new compounds being developed in the future.

A vast array of traditional medicines have been prepared from natural sources. If acidosis-dependent anti-cancer compounds are included in some traditional medicines used for cancer therapy for a long time, such compounds would be identified in in vitro assay using an acidic medium.

For the effective use of anti-cancer drugs in acidic cancer nests, it would be a good idea to develop nanoparticles in which a drug can be embedded at an alkaline pH and subsequently released under acidic conditions.

## 8. Conclusions

Anti-cancer drugs specific to acidic conditions have been detected in in vitro studies using an acidic medium. Chemotherapy using such drugs would ameliorate the serious conditions that develop with the current anti-cancer chemotherapy, because they would exhibit less of an effect on normal tissues, whose pH is slightly alkaline. Few drugs have been shown to exhibit high-level efficacy for attenuating cancer cell growth under acidic conditions until now. It is highly likely that new acidosis-dependent anti-cancer drugs will be developed, and cancer chemotherapy specific to acidic environments may be exploited as a standard protocol in the future. Some researchers are now focusing on studies of the efficacy of anti-cancer drugs under acidic conditions. The author hopes that many researchers will become interested in the “acidic world,” and that cancer chemotherapy will enter new areas.

## Figures and Tables

**Table 1:** Inhibitory efficacy of various compounds on cell proliferation under acidic conditions.

Efficacy	Compounds
Higher at acidic pH	lovastatin, cantharidin manumycin A *, ionomycin **
Lower at acidic pH	aphidicolin, vinblastine sulfate, paclitaxel, aclarubicin, trichostatin A, 17-AAG
No change at acidic pH	mitomycin C, bleomycin sulfate, methotrexate, daunorubicin, actinomycin D, camptothecin, etoposide, cytochalasin D, kenpaullone, cycloheximide, radicicol, cucurbitacin 1, hydroxyurea, doxorubicin

Inhibitory effect on HeLa cell proliferation was measured in media with an initial pH of 7.7 and 6.7 that decreased to 7.4 and 6.6, respectively, after 5 days of culture [14]. * mesothelioma cells [14], ** synovial cells [15].

**Table 2:** Effective doses of statins in mouse models.

Statins	Defined Daily Doses	Mouse Model	
		Effective Dose	Cancers (in literature)
Simvastatin	30 mg	50 mg/kg/day *	melanoma [36]
		50 mg/kg/day *	colon cancer [37]
Lovastatin	45 mg	10 mg/kg/day *	thyroid cancer [38]
		16 mg/kg/day *	ascites tumor [39]
Fluvastatin	60 mg	40 mg/kg/day *	leukemia [40]
		15 mg/kg/day **	breast cancer [41]
Atorvastatin	20 mg	16 mg/kg/day **	ascites tumor [42]
		10 mg/kg/day **	prostate cancer [43]

* oral, ** intraperitoneal.
